# Mixed Domain IR-Hyper-Raman Four Wave Mixing Spectroscopy

**DOI:** 10.1021/acs.jpclett.5c03188

**Published:** 2025-12-26

**Authors:** Ryan P. McDonnell, Daniel D. Kohler, John C. Wright

**Affiliations:** Department of Chemistry, 5228 University of Wisconsin-Madison, Madison, Wisconsin 53706, United States

## Abstract

Vibrational dephasing, relaxation, and spectra are useful probes of local environments in complex chemical systems. Here, we present the use of an infrared-hyper-Raman four-wave mixing spectroscopy, hyper difference frequency generation (HDFG) spectroscopy, as a method to resolve vibrational dephasing and spectra in the bulk. Coherent vibrations of dichloromethane and cyanocobalamin are resolved in both frequency and time. Tuning the two-photon interaction frequency toward an electronic resonance in cyanocobalamin enhances the vibrational signal, demonstrating electronic resonance enhancement through electron-vibration coupling mechanisms in cyanocobalamin. This work motivates use of mixed-domain HDFG to measure coherent vibrational spectra in both frequency and time and to extract electron-vibration coupling information in molecular systems.

## Graphical abstract

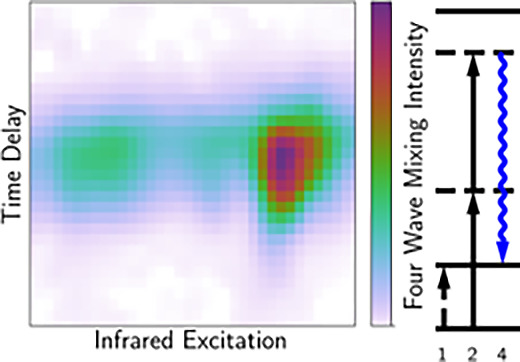

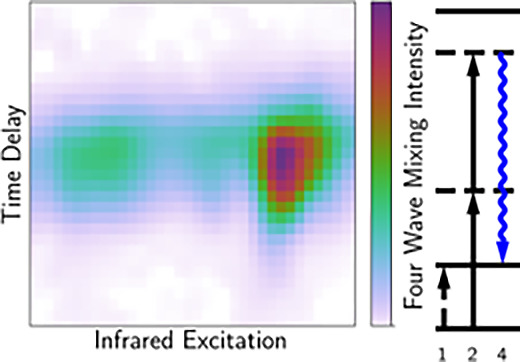

Dynamics and energetics of molecular vibrations are crucial for understanding the structure and local environment of biological and chemical systems.^1–30^ Several nonlinear spectroscopies and methods have been developed to measure vibrational dynamics and spectra by upconverting vibrational polarizations to the visible or near-IR region where detectors are more sensitive.^31–51^ Many of these methods use fully or partially coherent pathways to stimulate coherent output.^52,53^ If the coherent response is output in the infrared, it can be combined with other processes, such as sum-frequency generation, to enable visible detection by parametric upconversion, which creates a coherent beam in the visible.^54–56^ Instead of detecting coherent output, some upconversion methods are action detected, e.g., fluorescence or photocurrent detected.^32,47,48,50,57,58^ Some of these techniques can use the upconversion to interrogate the potential energy landscape of electronically excited states, vibronic coupling and other forms of electron-vibration coupling.^59–71^ The excitation schemes of some fully and partially coherent vibrational upconversion methods are presented in Figure 1 using wave mixing energy level (WMEL) diagrams.^72^ Of importance, the members of Figure 1 possess varying selection rules. For example, singly resonant vSFG demands a vibrational mode to be infrared and Raman active,^34^ singly resonant CARS demands a vibrational mode to be Raman active,^73^ and, under the Condon approximation, 2D-VE demands a ground state infrared active vibration have overlap with a coordinate on a one-photon accessible excited state.^66,67^

**Figure 1:**
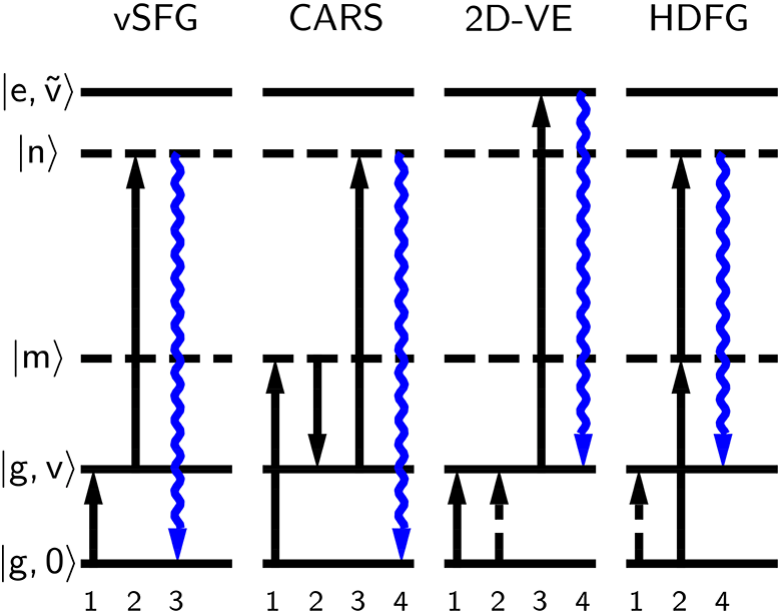
Wave mixing energy level (WMEL) diagrams^72^ of some vibrational up-conversion spectroscopies; from left to right: vibrational sum-frequency generation (vSFG), coherent anti-Stokes Raman spectroscopy (CARS), 2D vibrational-electronic spectroscopy (2D-VE), and HDFG.^34,62,66,69^ The solid (dashed) arrow indicates propagation of the ket (bra)-side density matrix element, and time is ordered from left to right; the numbers label the pulses, not time ordering. States are labeled using the adiabatic approximation:^74,75^ |*a*, *b*⟩ = |*a*)⊗|*b*⟩, where |*a*) is an electronic state and |*b*⟩ is a vibrational state.^76^ |g,0⟩ is the ground state, |*g*, *v*⟩ is a vibrational mode on |*g*), |m⟩ and |n⟩ are virtual states, and |e,ṽ⟩ is a vibrational mode on |*e*).

Hyper difference frequency generation (HDFG) spectroscopy^36,69,77^ (Figure 1, far right) is an underdeveloped and unique member of the vibrational upconversion spectroscopies. In HDFG, a tunable, mid-infrared pulse (ω_1_) excites a vibrational state, and a tunable, near-infrared pulse (ω_2_) stimulates Stokes hyper-Raman scattering to the same vibrational state.^69,77–80^ HDFG spectra are created by measuring the intensity of the **k**_4_ = – **k**_1_ + 2**k**_2_ output beam while the laser frequencies are scanned across vibrational resonances (ω_1_) and/or toward/across electronic (ω_2_) resonances.^36,69^ Under the electric dipole approximation, the HDFG hyperpolarizability, γ^(3)^_HDFG_, of the HDFG process depicted in Figure 1 scales with both the hyper-Raman hyperpolarizability, β_*vg*_, and the infrared transition moment μ_*gv*_ (the tensor properties of these quantities are suppressed for simplicity):^69^

1 $$\gamma_{\mathrm{HDFG}}^{\left(3\right)}∼\beta_{vg}\mu_{gv}$$

where μ_*gv*_ = ⟨*g*,0|μ|*g*,*v*⟩and β_*vg*_ = ⟨*g*,*v*|β|*g*,0⟩. The corresponding third order HDFG susceptibility, χ_HDFG_^(3)^, scales with ⟨γ_HDFG_^(3)^⟩, where the brackets indicate orientational averaging.^69^ Unlike some members of Figure 1, HDFG is allowed for all infrared active vibrations because infrared active vibrations are hyper-Raman active.^81^ In HDFG, all interaction wavelengths are far longer than the emission wavelength, so HDFG does not suffer from Rayleigh scatter contamination, but could still be contaminated by multiphoton fluorescence. HDFG experimental setups are also highly compatible with typical vSFG setups, so experimentalists may easily interchange between bulk and surface vibrational studies.^69^ These features make HDFG a unique and complementary member of the vibrational up-conversion spectroscopies.

As a technique involving the notoriously weak hyper-Raman transition,^82,83^ HDFG vibrational signals compete with nonresonant background and/or other nonlinear processes,^84–86^ which presents a significant barrier to sensitivity. The nonresonant background typically dictates the detection limits of nonlinear spectroscopies.^36,87^ In the first experimental implementation of singly resonant HDFG using nanosecond pulses, the exemplary IR active CH stretches in long-chain hydrocarbons exhibited a resonant susceptibility approximately an order of magnitude larger than the nonresonant background susceptibility.^36,77,84,88^ While this shows that HDFG vibrational spectroscopy is viable, the nonresonant background levels suggests applications are limited to concentrated samples (molar dilution factors of ∼100:1 or smaller) and relatively strong IR modes. It is thus important to develop HDFG techniques that suppress nonresonant background. Similar to nonlinear spectroscopies involving Raman transitions, such as vSFG and CARS, pulse durations, delays, and envelopes can be manipulated to isolate the vibrationally resonant contribution from the nonresonant contribution.^86,89–93^

In this letter, we demonstrate ultrafast HDFG spectroscopy as an upconversion method to investigate vibrational dynamics and electron-vibration coupling mechanisms. An ultrafast spectrometer, described more completely in the Supporting Information, uses separate optical parametric amplifiers (OPAs) to supply infrared (ω_1_) and near-infrared (ω_2_) pulses with approximately 1 ps pulsewidths. The pulses are routed on separate delay lines,^94^ which allows measurement of spectra as a function of delay time τ_12_, where τ_12_ ≡ τ_1_ – τ_2_ = −τ_21_; a negative value for τ_12_ indicates that ω_1_ arrives at the sample prior to ω_2_.^86^ The yaq and attune programs control the output frequency and delay lines of each OPA.^95,96^ By using infrared and near-infrared pulses with roughly 1 ps duration, the experiments reported here operate in the mixed domain, where the spectral bandwidth and temporal duration of the interaction pulses rival the line width and free induction lifetimes of vibrational modes, respectively.^90,97,98^ We examine vibrational resonances in a neat solvent (CH_2_Cl_2_) and thin films of a complex corrin ring system, cyanocobalamin (CNCbl). By delaying the two-photon interaction from the infrared interaction,^89^ vibrational spectra are drastically simplified, free from nonresonant background and fast vibrational modes. By also scanning the two-photon interaction frequency, we generate 2D frequency maps demonstrating electronic enhancement of vibrational resonances in cyanocobalamin, which demonstrates HDFG as a tool to investigate excited state potential energy surfaces and electron-vibration coupling mechanisms.^8,69,71^

We first illustrate HDFG vibrational spectra, and spectral simplification with the IR-two-photon interaction pulse delay, by examining CH_2_Cl_2_ in the ν­(CH) region. The experimental spectrum and its evolution with delay is presented in Figure 2a, corrected for absorption effects as described in the Supporting Information.^99^ The data shows two infrared features, which correspond to the well-known symmetric and asymmetric ν­(CH) modes, ν­(CH)_s_ and ν­(CH)_as_, at ∼2987 and 3053 cm^–1^, respectively. While CH stretches are strong infrared absorbers (cf. Figure S1), the CH_2_Cl_2_ CH stretches have weak hyper-Raman cross sections,^100^ suggesting mediocre HDFG activity through eq 1.

**Figure 2:**
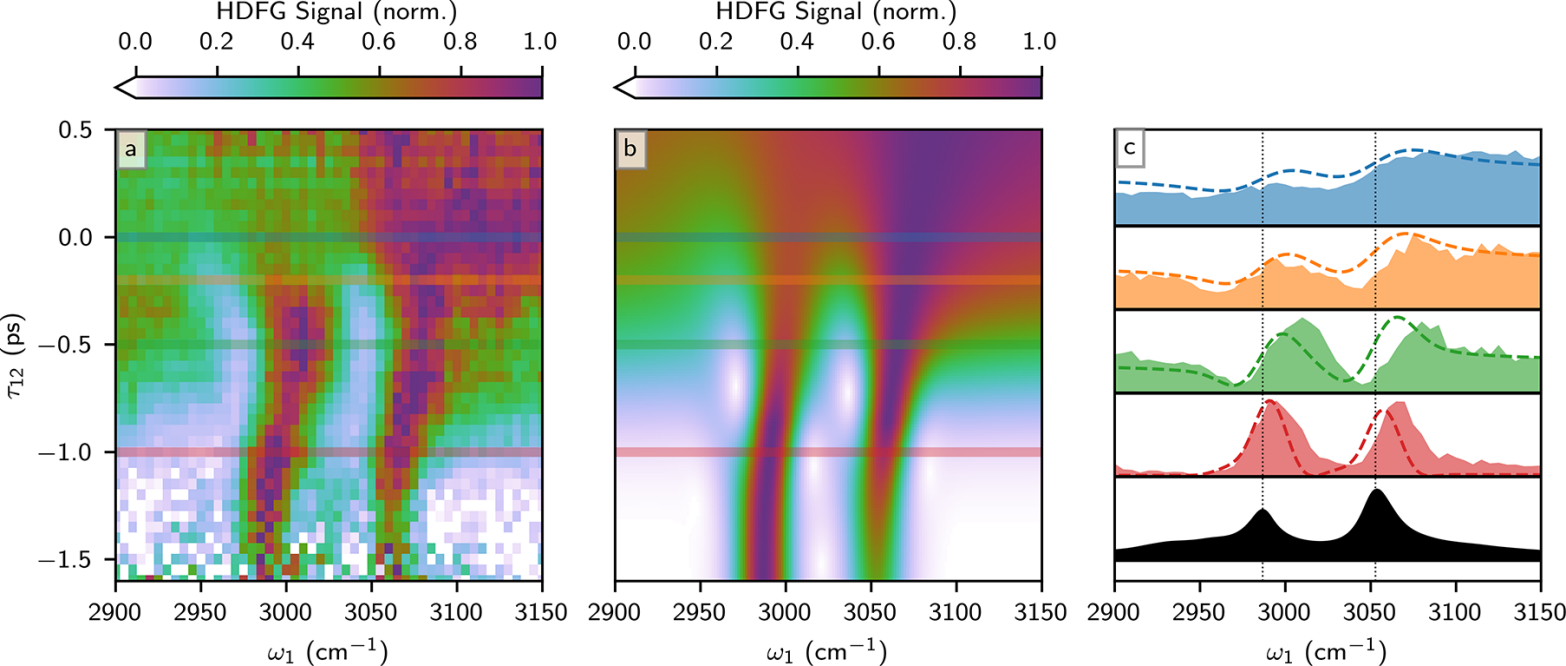
HDFG spectra of CH_2_Cl_2_ as a function of the IR frequency ω_1_ and two-photon interaction delay τ_12_. All spectra are normalized at each delay value to emphasize spectral evolution via pulse delay, reported as “HDFG Signal”. (a) 2D plot of HDFG of neat CH_2_Cl_2_ in the ν­(CH) region. The two-photon interaction was fixed at 2ω_2_ = 15200 cm^–1^. (b) Simulation of the CH_2_Cl_2_ HDFG response. The resonant response and nonresonant background are taken to have roughly equal amplitude in the simulation (see the Supporting Information for more details). (c) Comparison of HDFG spectra at selected delay values with the CH_2_Cl_2_ FT-IR spectrum (black). Filled spectra are the experimental results from (a) and dashed lines are simulations from (b). The plot colors correspond to the delays denoted by the lines in (a) and (b).

This middling HDFG activity is reflected in the spectrum at temporal pulse overlap (τ_12_ = 0). At this delay, the signal levels are large across the spectrum, even at ω_1_ colors far from ν­(CH) resonances, demonstrating a large nonresonant contribution. The ν­(CH) resonances have distorted lineshapes (blue trace, Figure 2c) resulting from interference with the nonresonant background.^77,87,99^ In the absence of the nonresonant background, the lineshapes would resemble the FTIR spectrum (black fill, Figure 2c) and peak at resonance. The large interference term changes the lineshapes to become dispersive, blueshifting the apparent peak positions from the actual positions.^99,101^ The extent of the blueshift will be similar to the pulse bandwidth or the vibrational resonance line width, whichever is larger.

As the arrival time of the two-photon interaction is delayed (τ_12_ < 0), the spectrum qualitatively changes because nonresonant and resonant interactions follow different dynamics.^102^ The nonresonant polarization follows the overlap of the interaction fields, while the resonant vibrational polarization persists due to free induction.^89^ As the two-photon interaction is delayed (negative τ_12_), both resonant and nonresonant signals decrease, but the resonant signal decreases slower, at a rate corresponding to its dephasing time. At delays exceeding the pulse duration (τ_12_ ≲ −1 ps), the hyper-Raman interaction overlaps in time with the free induction, and an effectively background-free vibrational spectrum is obtained.

The dynamics of the CH_2_Cl_2_ vibrational spectra in Figure 2a can be simulated using a standard perturbative expansion of the density matrix ρ by solving the Liouville-von Neumann equation

2 $$\mathrm{\rho ̇}=-\frac{i}{\hbar }\left[H\left(t\right),\rho \right]+{\mathrm{\rho ̇}}_{R}$$

under the electric dipole approximation in the interaction picture, as detailed in the Supporting Information;^103,104^ *H*(*t*) is the total system Hamiltonian and ρ_*R*_ is the relaxation density matrix. The resonant frequencies and dephasing rates of vibrational modes are extracted from the infrared spectrum (Figure S4). The amplitudes of the resonant and nonresonant contributions were fit to match the experimental data (Figure S2c, Table S1). The resulting simulation, shown in Figure 2b, has good agreement with the experimental data and re-enforces the role of the vibrational absorption on the HDFG spectrum and dynamics. The ν­(CH)_s_ resonance has a smaller HDFG susceptibility than ν­(CH)_as_ and is thus weaker at zero delay, but it is longer lived, so it becomes the larger feature when the HDFG interaction is delayed by more than 1 ps (τ_12_ < −1 ps). The simulation also shows small temporal undulations in intensity for IR frequencies in between the two vibrational resonances; these undulations arise from quantum beating between the two vibrational free inductions and repeat at a period of 1/(ν_s_ – ν_as_) ∼ 0.5 ps.^105,106^

The comparison of experiment and theory also illustrates the experimental challenge of choosing a τ_12_ value for evaluating spectra. For a sufficiently large delay value (here, around ∼1 ps), the nonresonant contribution dissipates and the HDFG IR spectrum converges to simple, nearly static vibrational features. Such delays comes at the cost of weakened signal, however, as the peak intensities dissipate as exp­(−2Γ_*vg*_τ_21_), where Γ_*vg*_ is the vibrational dephasing rate (see Figure S2 for representations of the data that show the signal dissipation with delay). In practice, the optimal delay balances spectral simplification with signal yield, and relative amplitudes will be influenced by their dephasing rate and quantum beating. Alternatively, note that data sets like those in Figure 2 evidence the vibrational parameters across both frequency and time, and multidimensional characterizations, like the simulation, offer a robust parameter extraction and sidestep the difficulties associated with picking a specific delay value.

While Figure 2 shows CH_2_Cl_2_ HDFG response can be resolved in both frequency and time, CH_2_Cl_2_ has electronic states that lie well outside the spectral range of our two-photon interaction. To assess the viability of HDFG response for vibronic study, we investigate preresonant HDFG response from a cyanocobalamin (CNCbl) thin film. CNCbl (Vitamin B_12_) is a biologically relevant molecule with a multitude of electronic and vibrational transitions.^107,108^ Particularly, CNCbl has a large vibrational density of states in the 1300–1650 cm^–1^ region and,^108,109^ due to its highly congested visible absorption spectrum,^107–111^ is an ideal target for investigating preresonant trends in the HDFG response. Monitoring HDFG intensity changes while scanning the two-photon interaction frequency, when the infrared pulse is resonant with a ground state vibrational mode, can inform on different types of electron-vibration coupling mechanisms.^69^ Since preresonance effects in Raman and hyper-Raman spectroscopy are important for interpreting vibronic coupling schemes,^8,112^ the ability to resolve preresonant HDFG from a CNCbl thin-film informs on the ability of HDFG to disentangle vibronic coupling and other types of electron-vibration coupling. Experimental confirmation of this ability would make HDFG a complementary technique to the IR-vis type of spectroscopies used to investigate electron-vibration coupling schemes.^46,64,65^

To identify whether HDFG is an appropriate tool for investigating electron-vibration coupling in CNCbl, a time-frequency scan at a static preresonant frequency is first performed to understand interference between the resonant and nonresonant response. Here, the CNCbl sample is dissolved in methanol and dispersed as a thin film on a microscope coverslip. By the time of experimentation, the methanol is evaporated, leaving the coverslip and CNCbl film as the sources of nonlinear response. The resultant CNCbl HDFG vibrational spectrum collected at 2ω_2_ = 15500 cm^–1^ (Figure 3a), an electronically preresonant frequency, shows several vibrational modes of CNCbl that dominate over the nonresonant contributions. The vibrationally resonant HDFG response likely dominates in CNCbl as compared to CH_2_Cl_2_ because there are only two sources of nonresonant contributions, and also maybe from electronic resonance enhancement from low lying CNCbl excited electronic states.

**Figure 3:**
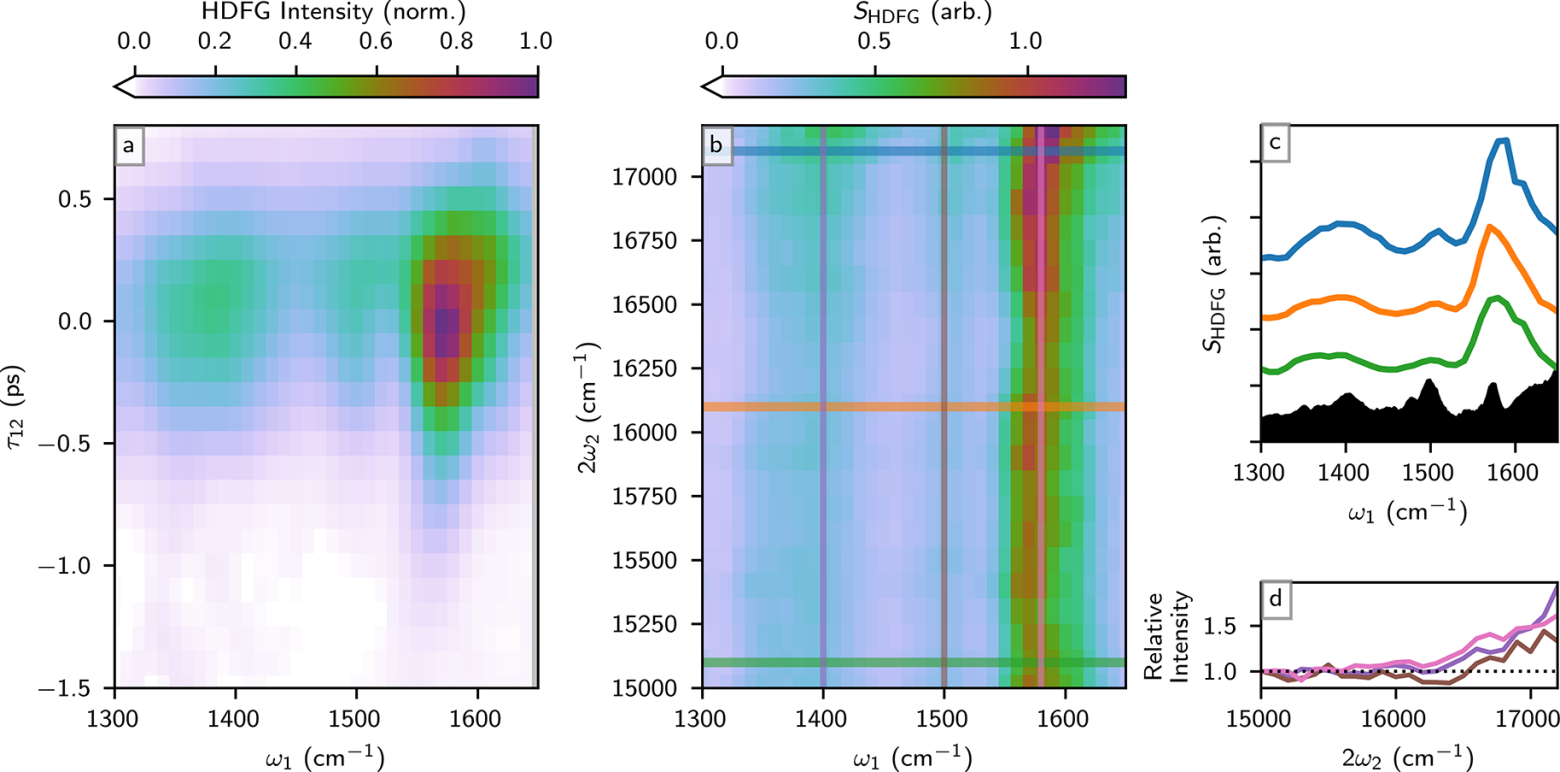
Evolution of the fingerprint spectra of cyanocobalamin (CNCbl) as the two-photon interaction approaches electronic resonance. (a) Cyanocobalamin HDFG spectra and their dependence on the two-photon interaction delay, τ_12_, where the two-photon interaction is set at 2ω_2_ = 15500 cm^–1^. The spectrum is slightly smoothed with a Kaiser window. (b) Evolution of the CNCbl fingerprint spectra as the two-photon interaction scans across preresonant frequencies. (c) Selected spectra in (b) are are shown as 1D plots and compared with the FTIR spectrum (black). Spectra in (b,c) are referenced to the output intensity at ω_1_ = 1800 cm^–1^, as described in the main text. Line colors correspond to the horizontal slices indicated in (b) and are arbitrarily offset to allow comparison. Also shown is the cyanocoblamin FT-IR spectrum (black). (d) Relative enhancement of 1400, 1500, and 1580 cm^–1^ modes across the two-photon interaction range explored. The enhancement in (d) is plotted relative to HDFG response at 2ω_2_ = 15000 cm^–1^, the reddest 2ω_2_ input frequency. Line colors in (d) correspond to the vertical slices indicated in (b).

Since the singly resonant vibrational HDFG response from CNCbl dominates over the nonresonant response, scanning the two-photon interaction toward electronic resonance should resolve potential electron-vibration coupling schemes in CNCbl. Recall that the HDFG hyperpolarizability depends upon the hyper-Raman hyperpolarizability and infrared transition dipole moment (eq 1).^61,69^ β is a frequency-dependent quantity that, under the Condon approximation, requires electronic transitions to be both one- and two-photon allowed.^61^ If a one- and two-photon allowed excited electronic state is offset relative to the ground state vibrational mode excited with the infrared pulse (i.e., nonzero Franck–Condon factors), then the HDFG output intensity will depend upon the two-photon interaction frequency under the Condon approximation.^61,69^ Non-Condon effects become important when the one and/or two-photon transition moments of an excited electronic state depend upon the vibrational coordinate excited with the infrared pulse. Since CNCbl has low symmetry, it is presumed that the electronic transitions are accessible by one- and two-photon transitions, which gives HDFG response under the Condon approximation.

By scanning 2ω_2_ toward the lowest lying electronic state in CNCbl (∼18200 cm^–1^) and tracking the relative intensity of specific vibrational modes, the coupling of CNCbl vibrational modes to the excited electronic state can be discerned. A two-dimensional frequency plot of the HDFG intensity as a function of the two-photon and infrared interactions, akin to spectra found in 2D-VE spectroscopy,^46,113^ can be generated to probe the electronically resonant onset in CNCbl. By measuring a three-dimensional data set to provide an intensity *I*_HDFG_ (ω_1_, 2ω_2_, τ_12_), the coherent dynamics and frequency dependent HDFG response can be discerned. Since the vibrationally resonant CNCbl response is much larger than any nonresonant response even when detuned from electronic resonance (Figure 3a), the time axis can be integrated to generate a two-dimensional plot *S*_HDFG_ (ω_1_, 2ω_2_)­

3 $$S_{\mathrm{HDFG}}\left(\omega_{1},2\omega_{2}\right)=\frac{\int d\tau_{12}\;I_{\mathrm{HDFG}}\left(\omega_{1},2\omega_{2},\tau_{12}\right)}{\int d\tau_{12}\;I_{\mathrm{HDFG}}\left(1800\;{\mathrm{cm}}^{-1},2\omega_{2},\tau_{12}\right)}$$

To account for frequency dependent power fluctuations, the two-dimensional frequency data in Figure 3 are referenced to the integrated response at (1800 cm^–1^, 2ω_2_) taken during the same scan (the denominator of eq 3). At 1800 cm^–1^, the infrared spectrum is void of any CNCbl vibrational resonances (Figure S2), so the HDFG spectrum collected at ω_1_ = 1800 cm^–1^ is void of vibrational resonances, serving as an internal standard. eq 3 also bins any frequency dependent fluctuations in the definition of zero-delay.^96^

The 2D HDFG frequency plot of CNCbl response (Figure 3b), plotted following eq 3, shows that CNCbl vibrational modes at ∼ 1400, 1500, and 1580 cm^–1^ grow in intensity as 2ω_2_ approaches a visible absorption feature. The modes at ∼1500 and 1580 cm^–1^ have been previously assigned and are related to corrin ring stretching modes in CNCbl,^108^ and may be involved in vibronic coupling mechanisms with the lowest lying excited electronic state of CNCbl.^111,114^ HDFG response at specific 2ω_2_ slices (Figure 3c) demonstrate how approaching electric resonance amplifies certain infrared active modes. Particularly, the large density of states ∼1400 cm^–1^ becomes enhanced and possesses more structure in the HDFG spectrum as 2ω_2_ approaches the electronic absorption onset in CNCbl (∼17000 cm^–1^). The growth in intensity in the 1500 and 1580 cm^–1^ modes are expected, as they have some involvement in vibronic coupling mechanisms in CNCbl.^108^ Tracking the specific intensity of the three identified vibrational modes as a function of the 2ω_2_ highlights a growth in intensity also near the CNCbl electronic absorption onset (Figure 3d). These infrared features grow in intensity because they are related to the vibrational modes of excited state surfaces in CNCbl. HDFG can therefore identify how infrared active, ground state vibrations are related to vibrational modes of excited state surfaces. Our ω_2_ spectral range was limited by our laser system, but by augmenting our OPAs with mixing crystals, the spectral range could be increased, so that the electronically resonant regions of CNCbl could be explored.

These results highlight frequency dependent HDFG response from a system with low symmetry (CNCbl). In low symmetry systems, many states can possess nonzero one- and two-photon absorption response, which gives hyper-Raman based spectroscopies response under the Condon approximation.^61^ For hyper-Raman transitions, non-Condon effects become important when an electronic transition is not simultaneously one and two-photon allowed.^61,115^ Such a constraint can become especially important in highly symmetric systems due to parity.^116^ However, even in systems that have relatively low symmetry, such as rhodamine 6G and other rhodamine dyes, non-Condon effects (e.g., Herzberg–Teller coupling^117^) have been shown to dominate spontaneous hyper-Raman response.^118^ A comparison of one- and two-photon absorption spectra is important for assessing whether non-Condon effects are important for systems measured by HDFG.^8,119^ While two-photon absorption spectra can be rare for most relevant species, the ease of collecting two-photon absorption spectra with a typical ultrafast pump–probe spectrometer will assist in assessing systems studied by HDFG,^120^ and whether HDFG response arises from Condon or non-Condon contributions.^69^ Since our work shows HDFG can be a viable probe of electron-vibration coupling, HDFG could prove useful as a probe of non-Condon effects in molecular systems, akin to spontaneous hyper-Raman scattering.

In summary, multidimensional hyper difference frequency generation (HDFG) spectroscopy has been demonstrated using ultrafast pulses and extended to the mixed-domain. By assessing HDFG response from two model systems, dichloromethane (CH_2_Cl_2_) and cyanocobalamin (CNCbl), it is clear that vibrational spectra and dephasing dynamics can be resolved using a mixed domain HDFG approach. The singly resonant HDFG response from CH_2_Cl_2_ and CNCbl, the latter of which has an unknown hyper-Raman response, is commensurate with their infrared spectra and easily interpreted. In the case of CNCbl, strong preresonant response is detected and the frequency dependent changes in HDFG intensity suggest an ability to resolve electron-vibration coupling schemes. Our results show that HDFG is a promising and complementary spectroscopy to other coherent vibrational and mixed vibrational-electronic spectroscopies.

## Supplementary Material



## Data Availability

The data and workup scripts that support this study are permissively licensed and available for reuse at 10.17605/OSF.IO/HJ328.

## Notes

Spectrometer description, supplementary FT-IR and HDFG spectra, simulation description (PDF)
