# Supplementary material for: Mixed Domain IR-Hyper-Raman Four Wave Mixing Spectroscopy
Source: J Phys Chem Lett. 2025 Dec 26;17(1):249–56. doi: 10.1021/acs.jpclett.5c03188 (PMC12794168; doi:10.1021/acs.jpclett.5c03188)
Supplement: Supplementary file 1 [file jz5c03188_si_001.pdf]

Supporting Information for: Mixed Domain IR-Hyper-Raman Four Wave Mixing Spectroscopy

Ryan P. McDonnell,[a] Daniel D. Kohler, and John C. Wright[b]
Department of Chemistry, University of Wisconsin - Madison, Madison, Wisconsin 53706, United States of America

CONTENTS

I. Experimental S2

II. Absorptive Effects Correction S2

III. Supplementary Spectra S3

IV. CH_2Cl_2 HDFG Simulation Details S5
A. Problem Formulation S5
B. Solution Form S6
C. Simulation Parameters S7

V. References S8

[a]Electronic mail: rpmcdonnell@wisc.edu
[b]Electronic mail: wright@chem.wisc.edu

I. EXPERIMENTAL

The ultrafast spectrometer, based upon that described elsewhere,[1] is driven by an 80 MHz Ti-Sapphire ultrafast oscillator (Spectra-Physics Tsunami) which creates 35 fs seed pulses. The seed is passed to a 1 kHz chirped pulse amplifier (Spectra-Physics Spitfire Ace) to create approximately 5 W output centered at $\sim$ 800 nm. A Fourier mask in the regenerative amplifier stretches the output pulses to roughly 1 ps duration. The resultant output was used to pump four independent optical parametric amplifiers (OPAs), two of which are relevant for this study. OPA1 (TOPAS-800, Light Conversion), referred to as ω_1, was tuned for difference frequency generation (DFG) with an external stage (NDFG "DF1", Light Conversion). OPA2 (OPA-800C, Spectra Physics), referred to as ω_2, was tuned in its signal arrangement. The beams were co-polarized. Grating and crystal positions in OPA1 were externally controlled using `WinTOPAS` (Light Conversion). To control frequency output from OPA2, the grating and BBO in OPA2 were motorized with 50 mm DC actuators (Ealing). The OPAs were independently tuned using the `attune` package, controlled by `yaq`.[2,3] Movable retroreflectors (Thorlabs) controlled by 50 mm DC actuators (Ealing) are used to measure coherence dynamics. The beamlines are enclosed and purged with dry air to minimize distortions from H_2O vapor absorption. All spectra are collected in a transmission geometry.

To record nonlinear spectra, ω_1 was stepped in 5 or 10 cm^{-1} increments (for CH_2Cl_2 and cyanocobalamin (CNCbl) measurements, respectively), ω_2 was stepped in 50 cm^{-1} increments (for CNCbl measurements), and the time delay was stepped in 50 or 100 fs increments (for CH_2Cl_2 and CNCbl measurements, respectively). The nonlinear output was isolated through chopping schemes and a set of spatial apertures, directed into a monochromator (Horiba Micro-HR) and homodyne detected with a photomultiplier tube (Hamamatsu H7422-20), recorded and converted to digital output using a data acquisition unit (National Instruments PCIe-6361). Third-order cross-correlations measuring the intensity of $2\mathbf{k}_2 - \mathbf{k}_1$ as a function of frequency and time-delay in CaF_2 were used to calibrate frequency-dependent temporal overlap.[3] Data collection is controlled by `Bluesky`.[4] HDFG intensities are normalized to OPA power *in-situ* by dividing the detected HDFG intensity by OPA output measured by home-built pyroelectric detectors.[5] All data were worked up using the open-source, publicly available scientific Python software stack.[6–8]

The dichloromethane sample (Sigma-Aldrich, suitable for HPLC, $>$ 99.8 %, CAS no. 75-09-2) was pressed between 180 µm sapphire (Swiss Jewel) windows with a pathlength of 120 µm set by a Teflon spacer. Cyanocobalamin [DOT Scientific Inc., CAS no. 68-19-9] was prepared as a thin film deposited on microscope cover glass. Cyanocobalamin was dissolved in methanol (Fisher Scientific, HPLC Grade, CAS no. 67-56-1) until the solution reached saturation ($\sim$ 10 mg CNCbl / 10 mL CH_3OH). All chemicals were used without further purification. The resultant solution was extracted with a syringe fitted with a membrane filter to remove undissolved cyanocobalamin and drop cast onto the coverglass. Cyanocobalamin thin film Fourier transform infrared (FT-IR) spectra were collected with a Bruker Tensor 27 FT-IR spectrometer. Dichloromethane FT-IR spectra were collected with a Bruker Alpha II compact FT-IR spectrometer.

II. ABSORPTIVE EFFECTS CORRECTION

The HDFG intensity scales as

$$I \sim M|\chi^{(3)}_{\text{HDFG}}|^2, \tag{S1}$$

where $\chi^{(3)}_{\text{HDFG}}$ is the HDFG susceptibility and M accounts for absorption and phase mismatch.[9] When the system only absorbs at ω_1 with no phase mismatch,

$$M(\omega_1) = \left(\frac{1 - 10^{-A(\omega_1)/2}}{A(\omega_1)\ln(10)/2}\right)^2, \tag{S2}$$

where $A(\omega_1)$ is the absorption at ω_1 convolved with a Gaussian to mimic broadening from the infrared pulse. By using the collected infrared absorption spectrum of CH_2Cl_2 (Figure S1) in Equation S2, the CH_2Cl_2 HDFG spectra are corrected for absorption effects by plotting as I_{HDFG}, where $I_{\text{HDFG}} = I/M$.

III. SUPPLEMENTARY SPECTRA

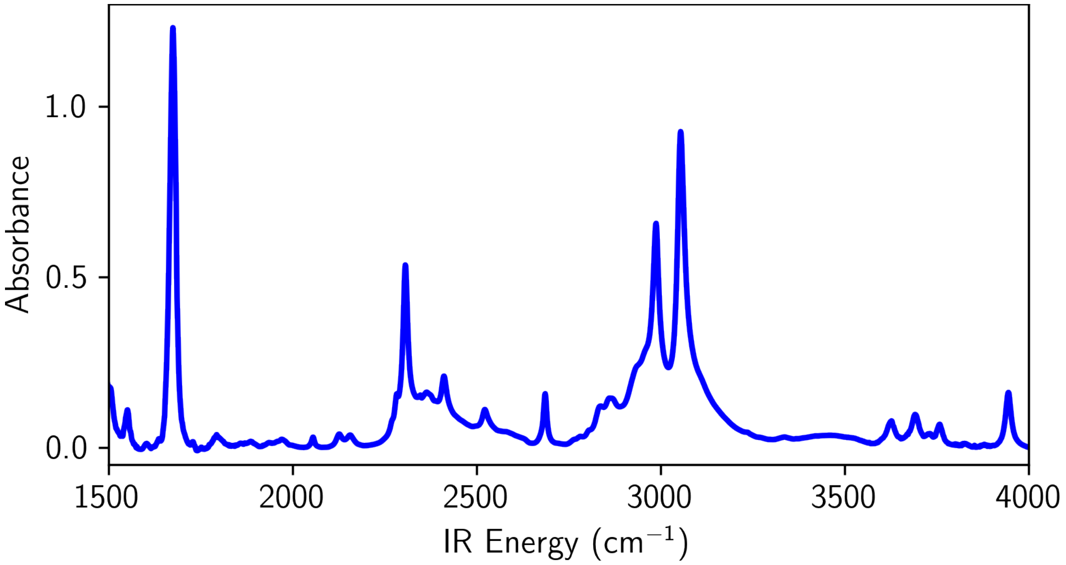

FIG. S1. FT-IR spectrum of neat CH_2Cl_2 in the sample cell used in the main text, collected at 293 K.

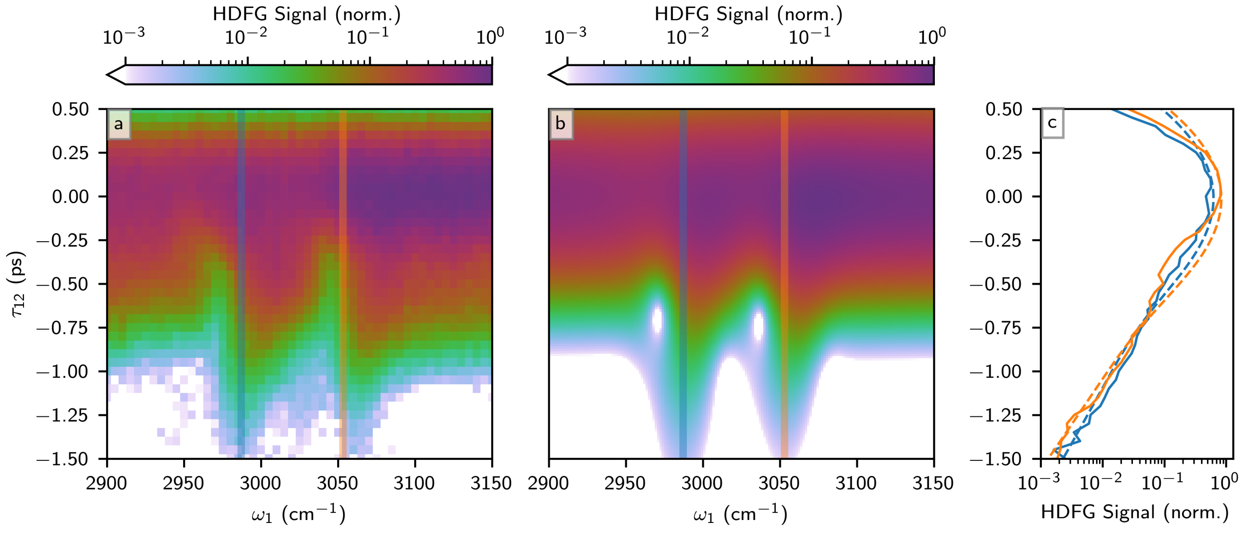

FIG. S2. Same data as Figure 2a of the main text, but rather than normalizing spectra at each delay, the data is instead normalized to the most intense feature in the 2D spectrum. The data are plotted on the intensity scale. A logarithmic scale is used to show the dynamic range of the data. (a) The experimental data. (b) The simulation. (c) Decay dynamics for resonant excitation at the $\nu(CH)_s$ (blue, solid) and $\nu(CH)_{as}$ (orange, solid) bands. Simulations at the same excitation colors are also shown in dashed lines. The two-photon interaction is fixed at $2\omega_2$ = 15200 cm^{-1}.

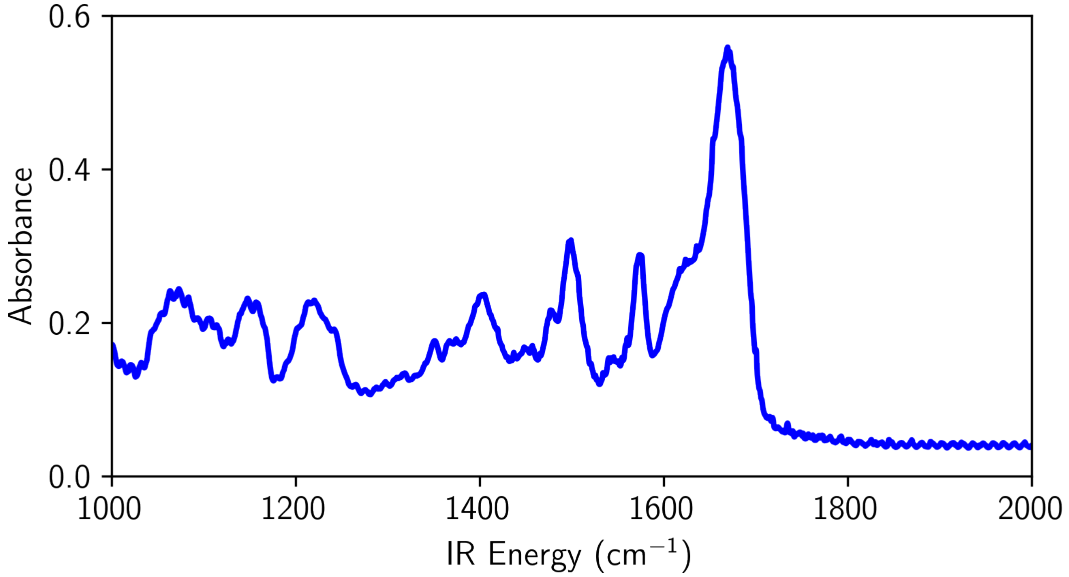

FIG. S3. FT-IR spectrum of the cyanocobalamin thin film used in the main text in the 1000 - 2000 cm^{-1} region, collected at 293 K. The fringes arise due to etaloning from the thin glass substrate.

IV. CH_2Cl_2 HDFG SIMULATION DETAILS

Here we derive the method used to describe the spectral and temporal dependence of HDFG signal in the main text. All tensor characteristics of the equations are suppressed.

A. Problem Formulation

The evolution of the density matrix is governed by the Liouville-von Neumann equation ($\hbar = 1$ throughout)

$$\dot{\rho} = -i[H_0 + V(t), \rho] + \dot{\rho}_R \tag{S3}$$

where H_0 is the static molecular Hamiltonian and $V(t)$ is the field-matter interaction.[10]

The field-matter interaction is described semi-classically under the electric dipole approximation as

$$V(t) = -\mu \sum_n E_n(t). \tag{S4}$$

Each excitation field E_n is decomposed into a envelope, $\tilde{E}_n(t)$, and a central frequency, ω_n: $E_n(t) = \frac{1}{2}\tilde{E}_n(t)\left(e^{-i\omega_n t} + e^{i\omega_n t}\right)$. Substituting in the Rabi frequency for the nth pulse as $\Omega^{(n)}(t) \equiv \mu \tilde{E}_n(t)$, we can write the elements of the time-dependent commutator as (summation over repeated index l implied):

$$[V(t), \rho]_{jk} = -\frac{1}{2}\sum_n \left(e^{i\omega_n t} + e^{-i\omega_n t}\right)\left(\Omega_{jl}^{(n)}(t)\rho_{lk} - \Omega_{lk}^{(n)}(t)\rho_{jl}\right), \tag{S5}$$

where $\Omega_{ab}^{(n)}(t) \equiv \langle a|\mu|b\rangle \tilde{E}_n(t) = \mu_{ab}\tilde{E}_n(t)$.

We use eigenfunctions of H_0 ($H_{0,jk} \equiv \langle j|H_0|k\rangle = \tilde{\omega}_k \delta_{jk}$, where δ is the Kronecker delta) to describe the density matrix, which simplifies the static commutator to (summation over repeated index l implied):

$$\begin{aligned}[H_0, \rho]_{jk} &= H_{0,jl}\rho_{lk} - \rho_{jl}H_{0,lk} \\ &= \tilde{\omega}_l \delta_{jl}\rho_{lk} - \rho_{jl}\tilde{\omega}_k \delta_{lk} \\ &= \omega_{jk}\rho_{jk},\end{aligned} \tag{S6}$$

where we have defined $\omega_{jk} \equiv \tilde{\omega}_j - \tilde{\omega}_k$. Following other treatments,[10–14] we will employ exponential dephasing ($\dot{\rho}_R = -\Gamma\rho$) to describe relaxation and decoherence, which is typically appropriate for vibrational excitation.[15] For an individual element of the density matrix, ρ_{jk}, the differential equation now reads (summation over repeated index l implied)

$$\dot{\rho}_{jk} = -i(\omega_{jk} - i\Gamma_{jk})\rho_{jk} + \frac{i}{2}\left(e^{i\omega t} + e^{-i\omega t}\right)\left(\Omega_{jl}\rho_{lk} - \Omega_{lk}\rho_{jl}\right). \tag{S7}$$

We now apply the perturbative expansion technique. That is, the propagation of the Liouville-von Neumann equation is broken into a sequence of propagations (Liouville pathways),[16,17] where, for each step in the sequence, only one electric field is used: $V(t) = -\mu E_n(t)$. Rigorously, the complete solution of the density matrix is given by summing over all pulse permutations, for all orders of interaction, and over all elements of the density matrix.[10,18,19] However, by spatially and spectrally gating our output to $\mathbf{k}_{\text{out}} = 2\mathbf{k}_2 - \mathbf{k}_1$ and $\omega_{\text{out}} = 2\omega_2 - \omega_1$, respectively, and neglecting higher order processes, we need only consider the lowest order pathways that scale as $E_2^2 E_1^*$.[16] The relevant HDFG Liouville pathway is then

$$\rho_{gg}^{(0)} \xrightarrow{1} \rho_{gv}^{(1)} \xrightarrow{2} \rho_{mv}^{(2)} \xrightarrow{2} \rho_{nv}^{(3)} \xRightarrow{\text{out}} \rho_{vv}^{(4)}, \tag{S8}$$

where the numbers above the arrows identify the interacting pulse and $\rho^{(k)}$ denotes a kth order density matrix element. Here, $|g\rangle$ is the ground state, $|v\rangle$ is a vibrational state, and $|m\rangle, |n\rangle$ are virtual states. Each transition represents a propagation of the Liouville-von Neumann equation where $V(t)$ includes just one of the excitation fields:[10,20]

$$\dot{\rho}^{(n)} = -i[H_0, \rho^{(n)}] - i[V(t), \rho^{(n-1)}] - \Gamma\rho^{(n)}. \tag{S9}$$

At this point we simplify Equation S9 using the interaction picture:[21] we define the interaction density matrix, $\tilde{\rho}$, as the trivial evolution ($V(t) = \Gamma = 0$) solution

$$\tilde{\rho}_{jk}(t) \equiv \rho_{jk}(t)e^{i\omega_{jk}t}. \tag{S10}$$

Then $\dot{\rho}_{jk} = \dot{\tilde{\rho}}_{jk}e^{-i\omega_{jk}t} - i\omega_{jk}\rho_{jk}$, which lets us write

$$\dot{\tilde{\rho}}_{jk}^{(n)} = -\Gamma_{jk}\tilde{\rho}_{jk}^{(n)} + \frac{i}{2}\left(\Omega_{jl}\tilde{\rho}_{lk}^{(n-1)}e^{i(\omega_{jl}\pm\omega)t} - \Omega_{lk}\tilde{\rho}_{jl}^{(n-1)}e^{i(\omega_{lk}\pm\omega)t}\right) \tag{S11}$$

If we are near resonance, we can take the rotating wave approximation and neglect one of the $\pm\omega$.[13]

B. Solution Form

We start with the first transition in pathway S8, where the vibrational coherence $\rho_{gv}^{(1)}$ is generated from $\rho_{gg}^{(0)}$ (taken to be unity) via electric field E_1. We take the rotating wave approximation and rewrite Equation S11 as

$$\dot{\tilde{\rho}}_{gv}^{(1)}(t,\omega_1) = -\Gamma_{gv}\tilde{\rho}_{gv}^{(1)} - \frac{i\mu_{gv}}{2}\tilde{E}(t)e^{i(\omega_{vg}-\omega_1)t}. \tag{S12}$$

A closed-form solution to Equation S12 can be realized by integrals in the time domain or the frequency domain; here, we use the frequency domain. Applying a Fourier transform to Equation S12,[a] one can solve for $P_{gv}(\omega;\zeta)$, the frequency-domain representation of $\tilde{\rho}_{gv}(t;\zeta)$:

$$P_{gv}(\omega,\omega_1) = \frac{\mu_{gv}}{\Gamma_{gv}+i\omega} \times \tilde{E}_\omega(\omega-(\omega_{vg}-\omega_1)), \tag{S13}$$

where $\tilde{E}_\omega(\omega)$ is the Fourier transform of $\tilde{E}(t)$,

$$\tilde{E}_\omega(\omega) = \int_{-\infty}^{\infty} \mathrm{d}t\, \tilde{E}(t)e^{-i\omega t}, \tag{S14}$$

otherwise known as the spectral envelope of the excitation pulse. Performing an inverse Fourier transform on Equation S13, we arrive at the solution as a spectral convolution:

$$\tilde{\rho}_{gv}^{(1)}(t,\omega_1) = \mu_{gv}\int_{-\infty}^{\infty} \mathrm{d}\omega\, \tilde{E}_\omega(\omega_{vg}-\omega_1-\omega)r(\omega,t), \tag{S15}$$

$$r(\omega,t) \equiv \frac{e^{i\omega t}}{\Gamma_{gv}+i\omega}. \tag{S16}$$

To generate the third order coherence, the second electric field interacts twice with virtual states (i.e., nonresonant excitation). By invoking the Placzek approximation,[23,24] so that the two-photon interaction occurs instantaneously and is invariant with respect to the frequency of the second excitation field,

$$\rho_{nv}^{(3)}(\omega_1,\tau) \sim \tilde{E}^2(\tau-t)\tilde{\rho}_{gv}^{(1)}(t;\omega_1)e^{-i(2\omega_2-\omega_{vg})t}. \tag{S17}$$

For the non-resonant background contribution, $\rho_{\mathrm{NR}}^{(3)}$, the first transition is also taken to be instantaneous and frequency invariant. This gives the time dependence

$$\rho_{\mathrm{NR}}^{(3)}(\tau) \sim \tilde{E}_2^2(\tau-t)\tilde{E}_1(t)e^{-i(2\omega_2-\omega_1)t}. \tag{S18}$$

The third-order polarization is

$$\begin{aligned} P^{(3)}(t;\omega_1,\tau) &= \mathrm{Tr}(\mu\rho^{(3)}(\omega_1,\tau)) \\ &= \beta_{vg}\tilde{E}^2(\tau-t)\tilde{\rho}_{gv}^{(1)}(t;\omega_1)e^{-i(2\omega_2-\omega_{vg})t} \\ &\quad + Ae^{i\phi}\tilde{E}_2^2(\tau-t)\tilde{E}_1(t)e^{-i(2\omega_2-\omega_1)t}, \end{aligned} \tag{S19}$$

where we have introduced proportionality factors A for the non-resonant contribution and $\beta_{vg} \equiv \langle v|\beta|g\rangle$ for the resonant contribution, respectively, and ϕ is a phase factor. The nonlinear polarization described by Equation S19 is then used as a source in the Maxwell equations to generate an output $E_{\mathrm{HDFG}}(t;\omega_1,\tau) \sim P^{(3)}(t;\omega_1,\tau)$,[25–27] assuming a slowly varying amplitude.[28,29] The HDFG envelope at frequency $2\omega_2-\omega_1$ can then be calculated via

$$\begin{aligned} E_{\mathrm{HDFG}}(2\omega_2-\omega_1,\tau) &\sim \int \mathrm{d}t\, P^{(3)}(t)e^{i(2\omega_2-\omega_1)t} \\ &= \beta_{vg}\int \mathrm{d}t\, \tilde{E}^2(\tau-t)\tilde{\rho}_{gv}^{(1)}(t;\omega_1)e^{-i(\omega_1-\omega_{vg})t} \\ &\quad + Ae^{i\phi}\int \mathrm{d}t\, \tilde{E}_2^2(\tau-t)\tilde{E}_1(t). \end{aligned} \tag{S20}$$

The homodyne detected HDFG response is then

$$I_{\mathrm{HDFG}} \sim \left|E_{\mathrm{HDFG}}(2\omega_2-\omega_1,\tau)\right|^2. \tag{S21}$$

---

[a] Relevant properties of the Fourier transform can be found in §4.8 and the end of §4 in *Morse and Feshbach*.[22]

C. Simulation Parameters

We used Gaussian functions to describe the temporal envelopes of the excitation fields:

$$\tilde{E}(t) = E_0 \exp\left(-\frac{t^2}{2\sigma_t^2}\right). \tag{S22}$$

The spectral envelope, defined by Equation S14 is therefore also Gaussian:

$$\tilde{E}_\omega(\omega) = \sqrt{\frac{1}{\sigma_\omega^2}} E_0 \exp\left(-\frac{\omega^2}{2\sigma_\omega^2}\right), \tag{S23}$$

and the two widths are inversely related by $\sigma_t \sigma_\omega = 1$. A pulse width parameter of $\sigma_t = 0.3$ ps (or $\sigma_w/2\pi c = 18$ cm^{-1}) agreed well with experiment. The phase of the nonresonant background is taken to be $\phi = 0$. The other parameters used to generate Figure 2 of the main text can be found in Table S1.

TABLE S1. Vibrational resonance parameters used to simulate the CH_2Cl_2 HDFG spectrum in the main text.

	$\omega_{vg}/2\pi c$ (cm^{-1})	$\beta\mu/A$ (a.u.)	$\Gamma_{vg}/2\pi c$ (cm^{-1})	$\tau_{vg} = 1/\Gamma_{vg}$ (ps)
$\nu(CH)_s$	2987	0.8	9.65	0.55
$\nu(CH)_{as}$	3053	1.0	11.80	0.45

The dephasing dynamics and homogeneous spectral linewidths used in this simulation are in good agreement with the FT-IR spectra (Figure S4).

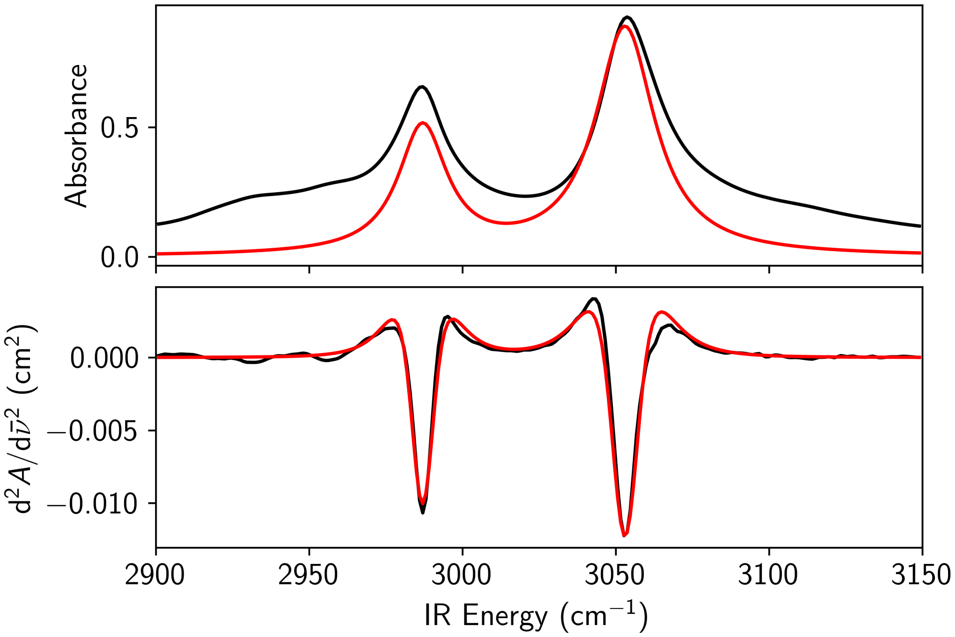

FIG. S4. Characterization of the CH_2Cl_2 ν(CH) modes. (a) Absorption spectrum in the range of the CH stretch modes. (b) The second derivative lineshapes, which minimizes broadband spectral components. In both figures, black is the experimental spectrum, and red represents a fit of the second derivative lineshape. The fit used Lorentzian lineshapes, whose linewidth parameters are found in Table S1.

V. REFERENCES

[1] J. D. Handali, K. F. Sunden, E. M. Kaufman, and J. C. Wright, Chemical Physics **512**, 13 (2018).
[2] K. F. Sunden, D. D. Kohler, K. A. Meyer, P. L. Cruz Parrilla, J. C. Wright, and B. J. Thompson, Review of Scientific Instruments **94**, 044707 (2023).
[3] K. F. Sunden, D. D. Kohler, R. P. McDonnell, D. J. Morrow, D. P. Lafayette II, E. M. Kaufman, J. M. Scheeler, J. D. Handali, K. J. Czech, B. J. Thompson, and J. C. Wright, Journal of Chemical Physics **163**, 014201 (2025).
[4] D. Allan, T. Caswell, S. Campbell, and M. Rakitin, Synchrotron Radiation News **32**, 19 (2019).
[5] K. A. Meyer and J. C. Wright, Analytical Chemistry **73**, 5020 (2001).
[6] S. van der Walt, S. C. Colbert, and G. Varoquaux, Computing in Science and Engineering **13**, 22 (2011).
[7] B. J. Thompson, K. F. Sunden, D. J. Morrow, D. D. Kohler, and J. C. Wright, Journal of Open Source Software **4**, 1141 (2019).
[8] P. Virtanen, R. Gommers, T. E. Oliphant, M. Haberland, T. Reddy, D. Cournapeau, E. Burovski, P. Peterson, W. Weckesser, J. Bright, S. J. van der Walt, M. Brett, J. Wilson, K. J. Millman, N. Mayorov, A. R. J. Nelson, E. Jones, R. Kern, E. Larson, C. J. Carey, İ. Polat, Y. Feng, E. W. Moore, J. VanderPlas, D. Laxalde, J. Perktold, R. Cimrman, I. Henriksen, E. A. Quintero, C. R. Harris, A. M. Archibald, A. H. Ribeiro, F. Pedregosa, P. van Mulbregt, and SciPy 1.0 Contributors, Nature Methods **17**, 261 (2020).
[9] R. J. Carlson and J. C. Wright, Applied Spectroscopy **43**, 1195 (1989).
[10] N. Bloembergen and Y. R. Shen, Physical Review **133**, A37 (1964).
[11] N. Bloembergen, H. Lotem, and R. T. Lynch Jr., Indian J. Pure Appl. Phys. **16**, 151 (1978).
[12] J. L. Oudar and Y. R. Shen, Phys. Rev. A **22**, 1141 (1980).
[13] D. Lee and A. C. Albrecht, A Unified View of Raman, Resonance Raman, and Fluorescence Spectroscopy (and their Analogues in Two-Photon Absorption), in *Advances in Infrared and Raman Spectroscopies*, edited by R. Clark and R. Hester (Wiley, New York, 1985) pp. 179–213.
[14] J. C. Wright, Journal of Physical Chemistry Letters **10**, 2767 (2019).
[15] J. Sung and R. J. Silbey, Journal of Chemical Physics **115**, 9266 (2001).
[16] S. Mukamel, Physical Review A **28**, 3480 (1983).
[17] J. C. Wright, R. J. Carlson, G. B. Hurst, J. K. Steehler, M. T. Riebe, B. B. Price, D. C. Nguyen, and S. H. Lee, International Reviews in Physical Chemistry **10**, 349 (1991).
[18] B. Dick and R. M. Hochstrasser, Chemical Physics **75**, 133 (1983).
[19] R. J. Carlson and J. C. Wright, Physical Review A **40**, 5092 (1989).
[20] Y. Prior, IEEE Journal of Quantum Electronics **20**, 37 (1984).
[21] C. J. Bordé, Density Matrix Equations and Diagrams for High Resolution Non-Linear Laser Spectroscopy: Application to Ramsey Fringes in the Optical Domain, in *Advances in Laser Spectroscopy*, edited by F. T. Arecchi, F. Strumia, and H. Walther (Springer US, Boston, MA, 1983) pp. 1–70.
[22] P. M. Morse and H. Feshbach, *Methods of Theoretical Physics* (McGraw-Hill, 1953).
[23] G. Placzek, Rayleigh-Streuung und Raman-Effekt, in *Handbuch der Radiologie*, Vol. 6, edited by E. Marx (Akademische Verlagsgesellschaft, Leipzig, 1934) p. 204, part 2.
[24] D. A. Long and L. Stanton, Proceedings of the Royal Society of London. A. Mathematical and Physical Sciences **318**, 441 (1970).
[25] J. A. Armstrong, N. Bloembergen, J. Ducuing, and P. S. Pershan, Physical Review **127**, 1918 (1962).
[26] N. Belabas and D. M. Jonas, J. Opt. Soc. Am. B **22**, 655 (2005).
[27] M. Cho, Chemical Reviews **108**, 1331 (2008).
[28] K. Park and M. Cho, Journal of Chemical Physics **109**, 10559 (1998).
[29] Y. R. Shen, *The Principles of Nonlinear Optics* (Wiley, 2003).
